# Khat Use: What Is the Problem and What Can Be Done?

**DOI:** 10.1155/2015/472302

**Published:** 2015-05-03

**Authors:** Yusuf Sheikh Omar, Anna Jenkins, Marieke van Regteren Altena, Harvey Tuck, Chris Hynan, Ahmed Tohow, Prem Chopra, David Castle

**Affiliations:** ^1^University of Melbourne Department of Psychiatry, St. Vincent's Hospital Melbourne, Fitzroy, VIC 3065, Australia; ^2^Victorian Transcultural Mental Health, St. Vincent's Hospital Melbourne, Fitzroy, VIC 3065, Australia; ^3^Northern Nexus, St. Vincent's Hospital Melbourne, Fitzroy, VIC 3065, Australia; ^4^Elite Enterprises Group Pty Ltd., Preston, VIC 3072, Australia; ^5^St. Vincent's Hospital Melbourne, Fitzroy, VIC 3065, Australia

## Abstract

The chewing of khat leaves is an established tradition in East Africa but is much less prevalent in other areas of the world and is mostly limited to Somali communities. However, our understanding of what constitutes problematic khat use in the Somali community in Victoria, Australia, is limited. The objectives of this study were to better understand the views of Somali community representatives and primary care practitioners regarding problematic khat use, to consider relevant harm minimisation strategies, and to develop resources to assist individuals with problematic khat use and their families. Qualitative research methods were used to investigate the experiences and perceptions of khat use among Somalis and mainstream primary care practitioners. Six focus groups were conducted with 37 members of the Somali community and 11 primary care practitioners. Thematic analysis was used to analyse transcripts. Various indicators of the problematic use of khat were identified, including adverse physical and mental health effects, social isolation, family breakdown, and neglect of social responsibilities. Potential harm minimisation strategies were identified including the adoption of health promotion through education, outreach to the community, and the use of universal harm minimisation strategies specifically tailored to khat use.

## 1. Introduction

Khat (*Catha edulis*) is a leaf cultivated in Kenya, Yemen, and Ethiopia [1, 2]. Khat contains the alkaloids cathine and cathinone which have amphetamine-like properties and has been used for centuries in many African countries for its euphoric effect and as a sanctioned cultural practice [2–4].

Frequent khat use in the long-term has been associated with various health effects, including oesophagitis, gastritis, duodenal ulcer, hepatic cirrhosis, autoimmune hepatitis, migraine, cerebral haemorrhage, pulmonary oedema, and myocardial infarction [5, 6]. With regard to psychotropic effects, khat may produce euphoria, increased confidence, and enhanced alertness [5, 7]. In terms of adverse psychological effects, khat can contribute to such conditions as depression, anxiety, mood instability, and mania. There have also been some case reports of drug-induced psychosis, and, in individuals with established psychotic disorders, khat use may lead to an increased risk of relapse [5, 8, 9]. Furthermore, the use of khat by individuals with a prior history of exposure to trauma has been associated with an increased risk of developing psychotic symptoms [3, 10–12].

In Somalia, khat is typically chewed by groups of men in culturally sanctioned gatherings [2, 3, 13]. Traditionally, khat consumption by women is considered socially unacceptable and is rarely used [14].

However, gender differences particularly in the Western countries have become less evident, with more women using it openly [3, 15]. In Australia, it has been reported that some Western teenagers also chew khat [16]. Globally, the use of khat has increased over the past few decades and the demographics of users are changing, with an increase in the proportion of younger users and also female users [17, 18].

With regard to the Somali community in Australia, 9,589 people identified as being of Somali ancestry according to the 2011 Australian Census data, with 54.8% of those living in the state of Victoria and 98.8% of individuals who identified as being born in Somalia live in the state capital city of Melbourne. The largest groups of Somalis in Melbourne are in the local government areas of Banyule, Moonee Valley, and Melbourne [19]. In Australia, awareness of the use of khat and its associated problems has been increasing, although the definition of problematic use, particularly when compared with other drugs, remains controversial [8, 20, 21].

In recent years there has been a significant increase in the amount of khat that is being imported into Australia where it is not locally cultivated on a large scale [8]. Although khat is categorised by the WHO as a drug of abuse [8, 19], it is not controlled at the UN level [22]. In the UK, a report by the UK Advisory Council on the Misuse of Drugs in 2005 recommended that khat use should be discouraged although it is not a prohibited substance [8]. In Australia, khat use has varying legal status across the different states and territories [16]. In Victoria, New South Wales, and Tasmania individuals with a licence may legally obtain up to five kilograms of khat per month for personal consumption [4, 16, 23]. In other states and territories, the possession, trade, and cultivation of khat are not legal [16].

Furthermore, indicators of problematic khat use are poorly defined. The culture of khat use in Western countries including Australia has been associated with adverse social conditions related to experiences of displacement and marginalisation of immigrant East African communities [4, 11, 21]. In the UK, it has been noted that disadvantaged and unemployed young Somalis are particularly vulnerable to problematic khat use in addition to other substances' abuse [23]. In Australia, an increased prevalence of cigarette smoking among khat users has been reported [16].

While knowledge and understanding of the problems associated with khat use has been increasing, there is a need to further understand how problematic khat use is defined and what strategies may minimise the potential harms associated with khat use, particularly in the Australian context.

## 2. Objectives

The objectives of this study were to

1. understand the views of the Australian Somali community representatives and primary care practitioners regarding the definition of problematic khat use;
2. identify harm minimisation strategies that have particular relevance to individuals who use khat and their families;
3. use these findings to develop resources that may assist individuals with problematic khat use and their families.

## 3. Method

This study used qualitative research methods to investigate the views and experiences of the Somali community and primary care practitioners. Banyule Community Health Centre (BCHC) was chosen as a location for this study as there is a significant Somali population in the centre's catchment area, the City of Banyule in Melbourne, Australia. Approval for the study was granted by St Vincent's Hospital Human Research and Ethics Committee (Reference: LRR Protocol 033/12).

Community participants were purposively selected through key members of the Somali Australian Friendship Association, as well as informal community networks that were identified as being impartial regarding the debate about khat use within the Somali community. Primary care practitioner participants were recruited through BCHC.

Six focus group interviews each lasting about three hours, comprising a total of 48 participants, were conducted representing Somali youth (*n* = 7), Somali elder men (*n* = 10), Somali elder women (*n* = 11), Somali community representatives (*n* = 9), and two primary care practitioners' focus groups from BCHC in West Heidelberg (*n* = 11). Data saturation was reached and hence no additional focus group interviews were required.

The participant focus groups of this study were culturally and linguistically homogeneous. Age differences were considered; youth were grouped separately from the community elders in order to avoid people feeling unable to comfortably express their views in the presence of community elders. The youth focus group consisted of 4 young men and 3 young women. Similarly, the community representative focus group consisted of 5 men and 4 women. For community elders, men and women were interviewed separately in Somali language. In Somali culture, women (particularly elder women) may not feel comfortable expressing their views in the presence of elder men.

Khat users were not specifically identified from non-khat users. This is because the use of khat is contentious amongst members of the Somali community, and hence the researchers did not intend to marginalise identified khat users in the study.

Interviews with primary care practitioners, youth, and community representatives were conducted in English by the bilingual primary researcher, and the focus groups with community elders were conducted in Somali language. Participant information sheets (in English) were distributed among participants prior to conducting the interviews. The primary researcher explained this information in Somali language for the community focus groups whose English was limited. The primary researcher was present in all focus groups and was accompanied by other members of the group acting as comoderators. Group interaction was encouraged in order to explore common and contrasting perspectives [24].

Three focus groups were held in Banyule Community Health Centre: one focus group was held in the Neighbourhood Renewal Community hub, one was held in a church used as a community centre, and one was held at a community centre in Coburg.

Participants were verbally informed that the results from the study may be shared in future for presentations at academic and community meetings, and could be published in the peer-reviewed literature. Participants were also assured that all data would be deidentified, the anonymity of each participant would be maintained, and all information would remain confidential.

Signed consent and completed surveys related to participants' demographic characteristics were obtained during focus group interviews. Interviews were recorded and transcribed verbatim. These data are stored at Victorian Transcultural Mental Health accessible only by researchers involved in this project.

The participants were coded in the following way according to the focus groups they were part of:

- EMFGP (elder male focus group participant);
- EFFGP (elder female focus group participant);
- Y (youth focus group participant):
  - YFGMP (youth focus group male participant),
  - YFGFP (youth focus group female participant);
- CR (community representative focus group participant):
  - CRFGMP (community representative focus group male participant),
  - CRFGFP (community representative focus group female participant);
- PPFGP (primary care practitioner focus group participant).

The transcripts from the focus group interviews were analysed using content analysis [25]. The domains identified in the semistructured interview with the focus groups were used as a guide for the initial grouping of key themes. In order to ascertain the views of participants, an “emic” perspective was adopted. Transcripts were analysed to group and refine common as well as contrasting themes. The themes identified were reviewed independently by the coauthors for verification.

## 4. Results

### 4.1. Health and Social Problems Associated with Khat Use

A full project report is available from Victorian Transcultural Mental Health (formerly Victorian Transcultural Psychiatry Unit) [26]. As summarised in Table 1, a variety of factors were identified as being associated with problematic khat use. An elderly participant in the EM focus group stated that the meaning of the term “khat” is “bahalkuqaaday” (be taken and eaten by a predator). Physical and mental health problems linked to khat use were prominent concerns articulated, particularly, by the elder female focus group participants.

“Many khat users were dealt with by psychiatrists… many developed sense of fear, they see something that doesn't exist… Therefore, I think khat harms people mentally particularly for those who add to other substances when they use khat.” (EFFGP7)

“… When people use it [khat] they have something called “thubab” which means when people chew [continuously] at least two or three days and they stop and they don't chew, they experience… hallucinations and they see things which obviously is not a normal thing.” (CRFGMP3)

All groups identified social problems associated with khat use, although the EF focus group provided most elaboration, emphasising family breakdown and problems created by khat users' loss of occupational and social role. Some participants argued that the problem is not khat itself but rather how Somali Australians use khat. Thus, the frequent use of khat in significant amounts was identified as being problematic.

The majority of participants in this study identify that “marfishes,” where khat is consumed, are unhygienic:

“Large institutionalized places of khat usages mainly restaurants…are not hygienically in the best shape…” (CRFGMP10)

Interestingly, some participants from EF claimed that places where women could go for khat use are cleaner and in better condition than men's places.

### 4.2. The Association of Khat Use with Other Legal or Illegal Substances

Most participants, with the exception of the youth focus group participants, underscored the association between khat use and cigarette use. CRFGMP9 stated, “Khat-chewing and cigarette go together. There is not a khat chewer who does not smoke or if there are, they are very few”. A former khat user from EM focus group explained how he and his associates used other substances like ginger and clove in order to enhance the flavour of khat.

Anecdotal associations of khat use with alcohol and marijuana were mentioned. EFFGP2 recounted, “Khat, drug, and alcohol are the same… [and] they lead to the same way of thinking and the same attitudes. Users of these substances attract each other.”

However, two male participants from community representatives rejected the view that khat is similar to alcohol and other drugs, suggesting that khat is a better alternative:

“I think they use khat as an alternative to other substances like marijuana or whatever so, in that sense it is actually good…there is no evidence or anecdotal evidence or lived experience where people combine illegal drugs with khat…” (CRFGMP10)

### 4.3. Khat Use and Social Integration

Many participants, mainly from the EM and EF focus groups, believed that khat use is a barrier to social integration and khat users are marginalised. As a result, they develop their own subculture and do not engage actively with non-khat user Somalis and the wider Australian community:

“It is impossible to chew khat and at the same time to mix and interact with other Somalis who do not chew even those from your own village let alone to interact with non-Somalis.” (EMFGP8)

Their poor engagement with others is also attributed to their altered sleep-wake cycle, the degree of use, and the isolating nature of locations where khat is chewed. CRFGMP7 expressed that khat users appear “to be hiding somewhere….”

### 4.4. Getting Help for Problematic Khat Use

Respondents provided diverse views regarding places where someone affected with khat use may get help. Some argued that Somalis do not identify khat use as a problem and subsequently do not seek help, while some acknowledged that stigma may also adversely influence help-seeking behaviour:

“…if a person is not identifying it as a problem, then they're not going to see it as needing help. Within the Somali community, I don't think we believe… in something called addiction.” (YFGFP6)

Some participants expressed the belief that khat users seek financial help from their extended families or social security benefits in order to purchase khat. A few, mostly young people, underlined that the community does not openly talk about problems associated with khat use and hence about how to help individuals and their families.

A number of participants acknowledged that some people with problematic khat use seek help from mosques and Islamic religious centres as an alternative to drug and alcohol counselling services:

“I've seen people who want to get clean then they turn to religion … and they start going to the mosque and you know when you start turning to religion, you know Islam it cleanses you so pretty much they give up their bad hobbies and you know what they do so that kind of becomes like an alternative kind of like a rehab for them…” (YFGFP5)

### 4.5. Suggested Strategies for Helping Individuals and Families Affected by Khat Use

A range of strategies to help individuals and families affected by khat use were identified by participants as noted in Table 2.

A few participants suggested that public awareness of khat could be modelled on the public awareness education programs for cigarette and alcohol. Some argued that because Somalis in general do not place as much value on the written word, education through word of mouth, verbal, and visual education via community radio and television may be worthwhile:

“…we are an oral society…so by educating people not with pamphlets and graphics and stuff like that but by Somali way; the certain way we communicate in our society…” (CRFGMP2)

Resolving the legal status of khat was also suggested by some participants as an important issue. However, some argued that illegalisation of khat could criminalize an entire generation of older men who use khat and that this step may increase the risk of marginalizing men who are already vulnerable. Hence harm minimisation was identified as a meaningful approach:

“We could also actually make a lot of men criminals, which is something I'm also against in that sense.” (YFGMP1)

“I think there should be a shift from the discussion of criminalisation to the harm-minimisation… if you remove that without a dialogue and discussion with the community, then it shifts to more an illicit drug.”

### 4.6. Perspectives of Primary Care Practitioners

A key theme that emerged from health care practitioners was the lack of experience and understanding in general, regarding khat. Only the single Somali participant (PPFGP5) identified that they had worked with a client who they were aware used khat. There was an identified need to further understand khat's relative impact and potential for dependence in order to better understand the nature of problematic khat use. As identified by the Somali participant, as a result of the “silent” nature of khat use in the community, problems associated with its use are not widely known:

“…it's a silent drug that's in use. And because it's silent the harms of it are probably also silent.” (PPFGP1)

### 4.7. Understanding When Khat Use Becomes Problematic

Despite their lack of specific knowledge, some PP mainstream participants suggested that khat could be compared to other substances of abuse and that it may become problematic when it is associated with impairment in people's level of functioning:

“…Being a stimulant I guess people are staying awake and then crashing out and sleeping so that's obviously affecting their family life…” (PPFGP2)

### 4.8. Care for Individuals with Problematic Khat Use

Despite the limited understanding of the specific issues related to khat use overall, one of the key themes that emerged was the need for mainstream drug and alcohol counselling services to be in a position to provide necessary assistance. Additionally, harm minimisation strategies used for other addictive substances were suggested to be helpful and could be applied effectively:

“…I'm not saying it's a perfect system…but that's the system that exists…what there is in these services is specialised knowledge about treatment of addiction…and treatment of problematic drug use…” (PPFGP1)

Mirroring the views of community members, a significant theme emerged relating to the potential risks of criminalizing culturally sanctioned practices, particularly without the involvement of the community:

“…if we made use of this drug illegal we'll be essentially turning all users of this drug into criminals straight away and you'd put them into a new system…” (PPFGP1)

### 4.9. Harm Minimisation

With respect to specific harm minimisation strategies, one of the key themes to emerge was the potential application of universal strategies with adaptation to khat use. A theme emerged that guidance may be found from considering strategies that have been successful in reducing the harms associated with tobacco, alcohol, cannabis, marijuana, amphetamine, and heroin use:

“…basic strategies like maybe reducing the amount that they're chewing, maybe reducing the frequency, if they're attending these group meetings every day for instance I don't know if that's feasible really or realistic that they could attend every second day…” (PPFGP7)

It was noted that there is potential for role models within the community to act as advocates for change and that there is the potential for one group member's behaviour to provide a catalyst to change in other members of the group:

“…sometimes there are a group of users and one of them will be having a lot of problems in their life but want to do something about it and will go and do something about it and then everyone else in that group will start to see that person making changes in their life, so it's kind of a bit of a snowball effect.” (PPFGP11)

Culturally sensitive intervention strategies were also suggested. In particular, it was noted that interventions may be more useful in a group format, rather than through individual therapy. Both community and PP participants acknowledged the need for more information and research, in order to understand the impact of khat use and to understand what specific strategies may effectively meet the needs of individuals with problematic khat use and their families.

## 5. Discussion

This study explored perceptions of khat and its perceived level of harm in the urban Australian context. It examined the complexities regarding possible interventions that may assist individuals with problematic khat use and their families. The qualitative methodology used allowed an elaboration of diverse views of the Somali community members.

Some respondents believed that khat use in Australia is an accepted cultural practice while others argued that it helps users escape from problems posed by the new environment and resettlement. A relationship between khat use and maintenance of Somali cultural identity amongst migrants in Australia has been identified in earlier studies [21]. The vast majority of interviewees from the Somali community in this study correlated khat use with many adversities including physical and mental health problems. Additionally, negative social impacts linked to problematic khat use, including family breakdown, poor social integration, and unemployment, were identified.

Furthermore, khat use was seen to be associated with use of other substances, although some respondents considered khat as less harmful than alcohol and other drugs. The vast majority agreed that Somalis do not identify khat use as problematic. However, khat users were identified as being marginalised, hence reinforcing the silent nature of the problem. The reservations noted regarding the potential implications of illegalising khat use and the marginalisation of users are consistent with findings by Feigin et al. [4].

PP participants noted the important role of mainstream services as for individuals with alcohol and other substance use disorders. This highlights the importance of education and training of primary care practitioners. As noted by Fitzgerald, there is an unmet need for education campaigns to reduce patterns of harmful consumption of khat among members of the African communities in Australia [8].

Integrating the perspectives of the Somali community participants and primary care practitioners, a range of strategies to help individuals and families affected by khat use were identified. These strategies include establishing culturally appropriate and sensitive services and using social resources, such as families, community networks, community services, education through the media, and employing other culturally relevant ways of transmitting knowledge and information such as word of mouth and visual strategies. Universal harm minimisation goals, such as reducing the amount of khat used and the time spent using khat, were recommended.

There are a number of limitations of this study. First, a group of problematic khat users was not specifically identified. Rather, the aim of this study was to seek the perspectives of a broad range of community members, including users and nonusers. Second, participants were drawn from a defined geographical region. Third, PP participants included drug and alcohol workers but did not include a broader range of primary care practitioners such as general practitioners or community nurses. Fourth, whilst saturation of perspectives was reached, a wider range of views may have been evident with a larger number of focus groups. Fifth, the data may have been enhanced if the focus groups were supplemented by face-to-face interviews with participants; this was not possible due to time constraints.

Notwithstanding limitations, the findings emphasise the need for a culturally informed and broader understanding of what constitutes problematic substance use. In this case, the study has facilitated development of information resources for individuals and their families and for primary care practitioners in the local community. In a broader context, the findings potentially have relevance to Somali communities living in other Western countries, as well as, more generally, to the use of lesser known substances favoured by specific cultural groups.

## 6. Conclusions

In conclusion, there is a need for a greater level of awareness of khat use in immigrant Somali communities and its associated problems including health effects, impairment in functioning, family disharmony, and marginalisation from society. Primary care practitioners are well placed to provide assistance to individuals with problematic khat use, employing harm minimisation strategies. A culturally sensitive approach is warranted and outreach primary care services have an important role in attempting to engage with members of the Somali community around this issue. Further training of primary care practitioners including drug and alcohol service practitioners is necessary to improve awareness of khat and its effects. Universal strategies of motivational interviewing and harm minimisation adapted to suit the cultural context, such as by involving members of the Somali community and family members, are warranted.

## Figures and Tables

**Table 1:** Problems associated with khat use.

Theme	Frequency response^*^
Social problems (including family breakdown and family violence, poor social integration, social isolation, and withdrawal)	All 48 out of 48: 100%

Adverse physical health effects (including gastrointestinal problems and dental problems)	Majority 36 out of 48: 75%

Adverse mental health effects (including mood instability, disturbed behaviour, and psychotic symptoms)	Majority 35 out of 48: 73%

Perpetuation of unemployment	Majority 28 out of 48: 58%

Neglect of social roles and responsibilities	Half 24 out of 48: 50%

Negative impact on level of functioning	Few 15 out of 48: 31%

Neglect of meaningful activities	Few 6 out of 48: 13%

^*^Few: less than half of the participants, Half: half of the participants, Majority: more than half of the participants, and All: all of the participants.

**Table 2:** Harm minimisation strategies for individuals with problematic khat use.

Theme	Frequency response^*^
Adopting a health promotion strategy through education	Majority 46 out of 48: 96%

Developing awareness of when khat use becomes problematic	Majority 41 out of 48: 85%

Focusing on established users, who are generally mature older men, and younger men who are at risk of problematic khat use as well as other substances' use	Majority 36 out of 48: 75%

Support through religious activities	Majority 34 out of 48: 71%

Increasing public awareness of potential harms associated with khat use through the use of written information and other various oral means	Half 24 out of 48: 50%

Development of community centres and social programs through which people who are at risk of khat use may be assisted to expand their social activities	Half 24 out of 48: 50%

Developing culturally sensitive intervention strategies using an outreach approach	Half 24 out of 48: 50%

Adopting universal harm minimization strategies specifically tailored to khat use	Few 9 out of 48: 19%

Encouraging mainstream drug and alcohol counselling services to provide assistance	Few 7 out of 48: 15%

Further discussion and debate regarding the legal status of khat	Few 4 out of 48: 8%

^*^Few: less than half of the participants, Half: half of the participants, Majority: more than half of the participants, and All: all of the participants.
